# Effects of preanalytical frozen storage time and temperature on screening coagulation tests and factors VIII and IX activity

**DOI:** 10.1038/s41598-017-11777-x

**Published:** 2017-09-22

**Authors:** Ying Zhao, Guofang Feng, Jie Zhang, Renjie Gong, Changming Cai, Limin Feng

**Affiliations:** 1 Department of Clinical Laboratory, The First Affiliated Hospital, College of Medicine, Zhejiang University, Qingchun Road No. 79, Hangzhou, 310003 China; 2 Women’s Hospital, School of Medicine, Zhejiang University, Xueshi Road No. 1, Hangzhou, 310006 China

## Abstract

Preanalytical quality control of blood samples is critical for tests of coagulation function and coagulation factor activity. Preanalytical storage time and temperature are the main variables. We investigated the effects of preanalytical frozen storage time and temperature on activated partial thromboplastin time (APTT), fibrinogen (Fbg), prothrombin time (PT)/international normalized ratio (INR), thrombin time (TT), factor VIII activity (FVIII:C), and factor IX activity (FIX:C) in frozen plasma. Samples (n = 144) were randomly and equally divided into four groups (storage at −80 °C or −20 °C) and analysed by CS5100 or CA7000 coagulation analysers. Baseline values and results after storage for 15 days, 1 month, 3 months, 6 months, and 1 year were measured after thawing. Mean percent changes and scatter plots were used to determine clinically relevant differences. The stabilities of coagulation tests and coagulation factor activities measured by the CS5100 system were consistent with those measured by the CA7000 system. At −80 °C, assessment samples of PT/INR, Fbg, and TT can be safely stored for 1 year, APTT for 6 months, and FVIII:C and FIX:C for 1 month. At −20 °C, samples of Fbg and TT can be stored for 1 year, PT/INR and FIX:C for 1 month, and APTT and FVIII:C for 15 days.

## Introduction

Preanalytical quality control of blood samples includes steps that can interfere with the measurement of coagulation function and coagulation factor activity^1^. Unsuitable samples can lead to unreliable haemorrhagic and thrombotic results, thereby affecting clinical decisions^1,2^. Preanalytical variables primarily include sample collection, transportation, centrifugation, assay method, storage time, and temperature^2,3^. With the development of a hierarchical medical system in China, some patients undergoing anticoagulant therapy or with cardiovascular, haematological, or hepatic diseases consult doctors in small hospitals, community hospitals, and clinics, and family doctors^4,5^. The samples collected from these patients for routine coagulation tests are sent to independent clinical laboratories (ICLs) or certain comprehensive hospitals for analysis, especially for assessment of factor VIII activity (FVIII:C) and factor IX activity (FIX:C)^5,6^. The time required to transport the samples to ICLs and comprehensive hospitals is often over 4 h, and therefore exceeds the time recommended by the Clinical and Laboratory Standards Institute (CLSI) (H21-A5)^7^. In addition, recent advances have determined that biobanks can facilitate the development of drugs and diagnostic tests for both public health and personalized medicine^8,9^. Biobank networks have been developed and established by collecting, authenticating, and preserving human and/or bacterial specimens^8^. Relevant data for ensuring long-term preservation and optimal use of samples for analysis of coagulation are lacking.

Our preliminary tests established the storage times and optimal temperatures for screening coagulation tests (activated partial thromboplastin time (APTT), fibrinogen (Fbg), prothrombin time/international normalized ratio (PT/INR), thrombin time (TT)) and D-dimers in asymptomatic individuals and hepatitis B patients, and those of FVIII:C and FIX:C in asymptomatic individuals, using whole blood samples and aliquoted fresh plasma^5,10,11^. We demonstrated that PT/INR, TT, and Fbg can be safely stored for ≤24 h at 4 °C and 25 °C in fresh separated plasma, APTT for ≤12 h at 4 °C and ≤8 h at 25 °C, and FVIII:C for ≤2 h and FIX:C for ≤4 h at 4 °C and 25 °C.

However, these results only partially meet the practical requirements, especially for factor activity measurement^10,11^. One of the primary reasons is that some laboratories choose to accumulate large numbers of samples for coagulation tests for simultaneous measurement to save on costs and assay time. In addition, samples are sometimes collected during non-operating hours of laboratories, and there are centralized laboratory measurements for external quality assessment, clinical trials, and multicentre studies^2,12–14^. We aimed to establish appropriate sample storage times and temperatures for screening coagulation tests and coagulation factor activity assays for the treatment and monitoring of oral anticoagulant therapy, haemophilia, liver diseases, and thrombotic diseases^15–19^.

Perioperative fresh-frozen plasma (FFP) contains a number of coagulation factors, and is often used in patients with coagulopathy who have multiple coagulation factor deficiencies and are actively bleeding. After thawing, FFP is also used for patients with major trauma and/or massive haemorrhage^20–22^. Kuta *et al*.^22^ and Lamboo *et al*.^23^ analysed the quality of coagulation factors immediately after thawing FFP, and found that FFP units contained adequate coagulation factor activities for maintaining haemostatic function. Therefore, using fresh plasma samples that were immediately centrifuged after collection before being aliquoted and frozen, we aimed to investigate whether storage temperature (−20 °C or −80 °C) and time affected coagulation function and coagulation factor activity, and whether any changes caused by delayed analysis resulted in clinically relevant differences. In addition, we aimed to establish our own acceptable storage temperature and time guidelines for frozen plasma. In the present study, we measured APTT, Fbg, PT/INR, TT, FVIII:C, and FIX:C in both fresh samples and frozen samples stored at −20 °C or –80 °C for 15 days, 1 month, 3 months, 6 months, and 1 year using Sysmex CS5100 and CA7000 coagulation analysers.

## Methods

### Subjects

The study included 144 asymptomatic individuals who visited the First Affiliated Hospital of Zhejiang University for a physical examination in November 2014. The 144 subjects comprised 72 males and 72 females with a median age of 43 years (range: 19–78 years).

### Ethics statement

This study was approved by the Ethics Committees of the First Affiliated Hospital of Zhejiang University. Subjects provided written informed consent for their samples to be used in the study according to the principles expressed in the Declaration of Helsinki.

### Assays

Venepunctures were performed in the morning following a 12-h fast. A 5.4-ml venous whole blood sample was collected from each patient into two 2.7-ml tubes containing 0.109 M sodium citrate as an anticoagulant (Becton Dickinson, Franklin Lakes, NJ, USA) at a blood-to-anticoagulant ratio of 9:1. The two samples from each patient were centrifuged (10 min, 3000 × *g*) to obtain fresh plasma without platelets and cells, and the fresh plasma samples from the two tubes were mixed in an empty tube. The fresh plasma was quickly aliquoted into six Eppendorf tubes composed of a nonactivating plastic and capped. The 144 samples were randomly divided into four groups: 36 samples were frozen at −20 °C, stored at −20 °C, and analysed with the CA7000 system; 36 samples were frozen at −80 °C, stored at −80 °C, and analysed with the CA7000 system; 36 samples were frozen at −20 °C, stored at −20 °C, and analysed with the CS5100 system; and 36 samples were frozen at −80 °C, stored at −80 °C were analysed with the CS5100 system. One aliquot for each patient was analysed immediately after sample collection to assess the baseline (0 day) values. After storage for 15 days, 1 month, 3 months, 6 months, and 1 year, respectively, at −20 °C or −80 °C, the remaining five aliquots were tested after thawing in a thermostatic water bath for 10 min at 37 °C. The samples were mixed six times by end-over-end inversions before testing. All testing was completed within 30 min after thawing. The samples were tested for APTT, PT/INR, TT, Fbg, FVIII:C, and FIX:C by a coagulation-based nephelometric method using the CA7000 system (Sysmex, Kobe, Japan) or a coagulation-based turbidimetric method using the CS5100 system (Sysmex), with the following reagents from Siemens (Sysmex, Marburg, Germany): Dade Actin activated cephaloplastin reagent (lot: 557211); Dade Thrombin reagent (lot: 538097); Thromborel S (lot: 545584); Test Thrombin reagent (lot: 43668); Coagulation factor VIII-deficient plasma (lot: 546588B); and Coagulation factor IX-deficient plasma (lot: 504102). The results are presented as s (PT, APTT, TT), % (FVIII:C, FIX:C), and g/L (Fbg).

## Statistical Analysis

The results for the screening coagulation tests and coagulation factor activities were presented as mean ± standard deviation when the data were normally distributed or median (range) when the data distribution were skewed. The statistical significance of differences among repeated-measure multiple groups or compared with baseline values was assessed by repeated-measures analysis of variance or the Friedman test (Wilcoxon test) as appropriate. To assess the stability of the coagulation tests and coagulation factor activities, percent changes compared with baseline values were calculated [(result at storage time X − result at baseline)/result at baseline] and averaged for each time point. According to our previous study^10^ and the study by van Geest-Daalderop *et al*.^24^, a clinically relevant difference was defined as a mean percent change of >10%. If the number of samples with >10% change was less than 25% of the total sample number, the effect was considered moderate. If more than 25% of the samples had a change of >10%, the effect was considered large. Plots of the differences in percent changes were constructed with the percent changes of samples on the y-axis and the baseline values on the x-axis. The plots were visually inspected to determine the underlying variability characteristics of the relationship between storage time and baseline values. Individual sample differences were also observed. Two dotted lines were drawn in the figures to represent plus or minus 10% changes. P < 0.05 was considered statistically significant. Statistical analyses were performed using SPSS version 22 (SPSS, Chicago, IL, USA).

## Results

### Stability studies

Table 1 shows the results and statistical differences in the screening coagulation tests and coagulation factor activities of the plasma samples stored for 15 days, 1 month, 3 months, 6 months, and 1 year at −20 °C or −80 °C and analysed by the CS5100 and CA7000 systems, compared with the baseline values (0 h). Table 2 shows the mean percent changes and the stabilities of PT/INR, APTT, Fbg, TT, FVIII:C, and FIX:C when the samples were stored under the aforementioned conditions and analysed by the CS5100 and CA7000 systems. The mean changes of Fbg and TT were less than 10% for all time points and the mean percent change of FIX:C was less than 10% after sample storage for 1 month, regardless of storage temperature. The mean percent changes of PT/INR were less than 10% after sample storage for 1 year at −80 °C and 1 month at −20 °C. Although the mean percent changes of APTT after 1 month at −20 °C were 9.77% for the CS5100 system and 9.60% for the CA7000 system, changes of >10% in individual samples occurred in >25% of the samples. The mean percent changes of FVIII:C were less than 10% after sample storage for 1 month at −80 °C and 15 days at −20 °C. Therefore, assessment samples for Fbg, TT, and FIX:C can be safely stored for 1 year, 1 year, and 1 month regardless of storage temperature, samples of PT/INR for 1 year, samples of APTT for 6 months, and samples of FVIII:C for 1 month at −80 °C, while samples of PT/INR and FIX:C can be stored for 1 month and samples of APTT and FVIII:C for 15 days at −20 °C. Meanwhile, we found that the stability times based on different analysis principles (CA7000: a coagulation-based nephelometric method; CS5100: a coagulation-based turbidimetric method) were consistent.

**Table 1:** Results for PT/INR, APTT, Fbg, TT, FVIII:C, and FIX:C detected by the CS5100 and CA7000 systems after storage for 15 days, 1 month, 3 months, 6 months, and 1 year.

Terms	Baseline	15 days	1 month	3 months	6 months	1 year	*P*
**CS5100/−80 °C**							
PT (s)	10.8(9.9–16.1)	11.0(10–16.3)*	11.0(10.1–16.6)*	11.1(10.2–16.8)*	10.9(10–16.8)*	11.3(10.3–17.1)*	<0.001
INR	0.94(0.86–1.39)	0.96(0.87–1.40)*	0.96(0.88–1.43)*	0.97(0.89–1.44)*	0.95(0.87–1.44)*	0.94(0.85–1.47)	<0.001
APTT (s)	30.8 ± 3.8	30.4 ± 6.0*	31.7 ± 6.3*	32.3 ± 6.6*	32.6 ± 6.9*	34.7 ± 7.0*	<0.001
TT (s)	17.4 ± 1.2	17.6 ± 1.1*	17.7 ± 1.3*	17.7 ± 1.1*	17.9 ± 1.2*	18.1 ± 1.0*	<0.001
Fbg (g/L)	3.13 ± 1.17	3.15 ± 1.17*	3.13 ± 1.18	3.12 ± 1.18	3.14 ± 1.17*	3.15 ± 1.17	<0.001
FVIII:C (%)	129.7(77.3–325.7)	125.6(77.3–311.4)	123.5(75.4–269.0)*	115.0(69.8–302.8)*	112.9(67.2–297.7)*	112.1(68.6–281.3)*	<0.001
FIX:C (%)	104.5 ± 24.6	100.3 ± 23.5*	96.2 ± 22.7*	89.4 ± 22.4*	78.2 ± 22.0*	74.5 ± 19.2*	<0.001
**CS5100/−20 °C**							
PT (s)	10.9(9.6–12)	11.4(9.9–12.3)*	11.4(9.9–12.4)*	12.4(10.5–14.4)*	12(11.1–14.5)*	14.2(11.7–17.1)*	<0.001
INR	0.95(0.84–1.04)	0.99(0.86–1.07)*	0.99(0.86–1.08)*	1.08(0.92–1.24)*	1.04(0.97–1.24)*	1.21(0.98–1.47)	<0.001
APTT (s)	31.1 ± 3.8	32.7 ± 4.6*	34.2 ± 5.1*	36.5 ± 6.0*	36.9 ± 5.7*	44.0 ± 7.4*	<0.001
TT (s)	17.6 ± 1.0	17.7 ± 1.1	18.0 ± 1.1*	18.0 ± 1.0*	18.0 ± 0.8*	18.5 ± 1.35*	<0.001
Fbg (g/L)	2.89 ± 0.67	2.89 ± 0.66	2.87 ± 0.66	2.90 ± 0.67	2.91 ± 0.67*	2.89 ± 0.65	<0.001
FVIII:C (%)	109.7(72.2–234.4)	107.7(68.7–237.1)*	93.4(58.8–203)*	93.3(62.3–213.7)*	82.3(46.6–204.5)*	67.5(41.6–158.9)*	<0.001
FIX:C (%)	92.7 ± 16.5	86.9 ± 15.0*	86.8 ± 14.9*	79.5 ± 13.6*	68.3 ± 13.1*	64.3 ± 11.9*	<0.001
**CA7000/−80 °C**							
PT (s)	11.2(10.1–12.7)	11.3(10.1–13)	11.1(10–12.9)	11.1(10.1–12.8)	11.4(10.3–13.3)*	11.8(10.7–13.4)*	<0.001
INR	0.98(0.88–1.1)	0.98(0.88–1.12)	0.97(0.87–1.12)	0.96(0.87–1.13)	0.99(0.9–1.15)*	1.03(0.93–1.17)*	<0.001
APTT (s)	28.9 ± 4.0	30.5 ± 4.8*	30.1 ± 4.6*	30.3 ± 4.6*	31.6 ± 5.0*	34.3 ± 5.2*	<0.001
TT (s)	19.3 ± 0.9	19.3 ± 0.9	19.5 ± 0.9*	19.8 ± 1.2*	19.8 ± 1.4*	20.4 ± 1.4*	<0.001
Fbg (g/L)	2.87 ± 0.71	2.90 ± 0.73*	2.90 ± 0.72*	2.87 ± 0.72	2.90 ± 0.72*	2.91 ± 0.72*	<0.001
FVIII:C (%)	117.1(62.5–223.1)	110.7(66.7–184.0)*	106.2(55.6–189.3)*	99.7(58.5–164.4)*	99.0(60.8–163.0)*	96.4(52.6–177.2)*	<0.001
FIX:C (%)	93.8 ± 22.1	91.8 ± 20.0*	88.6 ± 20.0*	82.1 ± 18.8*	64.5 ± 15.4*	61.6 ± 14.7*	<0.001
**CA7000/−20 °C**							
PT (s)	10.9(9.9–13.5)	11.7(10.5–13.9)*	11.8(10.6–14.2)*	12.4(11.1–14.8)*	13.7(11.9–16.9)*	14.4(12.6–17.7)*	<0.001
INR	0.95(0.87–1.17)	1.02(0.92–1.2)*	1.02(0.92–1.23)*	1.08(0.97–1.27)*	1.19(1.11–1.47)*	1.28(1.11–1.61)*	<0.001
APTT (s)	28.1 ± 5.2	30.3 ± 5.8*	30.8 ± 6.0*	31.6 ± 6.4*	34.5 ± 6.9*	41.1 ± 8.3*	<0.001
TT (s)	19.5 ± 0.9	19.6 ± 1.0*	19.7 ± 1.1*	19.8 ± 1.0*	19.7 ± 1.3*	21.0 ± 2.0*	<0.001
Fbg (g/L)	2.89 ± 0.68	2.87 ± 0.67	2.87 ± 0.67	2.89 ± 0.68	2.91 ± 0.67	2.88 ± 0.67	<0.001
FVIII:C (%)	118.6(61.2–168.6)	111.8(43.4–172.3)*	105.4(53.6–147.6)*	97.9(48.2–145.1)*	92.2(45.4–166)*	74.5(30.4–107.2)*	<0.001
FIX:C (%)	95.2 ± 20.0	91.3 ± 18.3*	87.6 ± 18.5*	77.7 ± 17.5*	62.0 ± 12.2*	59.7 ± 13.2*	<0.001

*p < 0.05 compared with baseline values.

Abbreviations: APTT, activated partial thromboplastin time; Fbg, fibrinogen; PT, prothrombin time; INR, international normalized ratio; TT, thrombin time; FVIII:C, factor VIII activity; and FIX:C, factor IX activity.

**Table 2:** Mean percent changes of PT/INR, APTT, Fbg, TT, FVIII:C, and FIX:C detected by the CS5100 and CA7000 systems after storage for 15 days, 1 month, 3 months, 6 months, and 1 year compared with the baseline values.

Terms	15 days		1 month		3 months		6 months		1 year		Acceptable Time (h)	
CS5100	−80 °C	−20 °C	−80 °C	−20 °C	−80 °C	−20 °C	−80 °C	−20 °C	−80 °C	−20 °C	−80 °C	−20 °C
PT (s)	1.38	4.29	2.24	4.75	2.42	12.36*	1.71	11.11*	4.09	30.73*	1 year	1 month
INR	2.26	4.22	1.41	4.69	2.48	12.07*	1.68	10.75*	0.36	28.2*	1 year	1 month
APTT (s)	−1.3	4.96	2.63	9.77*	4.62	17.14*	5.65	18.39*	12.65*	41.53*	6 months	15 days
TT (s)	1.23	0.27	1.78	2.01	1.74	2	3.12	2.51	4.4	4.71	1 year	1 year
Fbg (g/L)	0.68	0.11	−0.05	−0.37	−0.26	0.45	0.69	0.99	0.71	0.48	1 year	1 year
FVIII:C (%)	−1.52	−2.94	−3.79	−16.58*	−10.1*	−12.6*	−11.33*	−24.52*	−11.98*	−37.86*	1 month	15 days
FIX:C (%)	−3.97	−5.96	−7.9	−6.19	−14.68*	−14*	−25.84*	26.4*	−29.04*	−30.61*	1 month	1 month
**CA7000**												
PT (s)	0.42	6.17	−0.44	7.81	−0.14	13.23*	2.08	24.82*	5.61	33.33*	1 year	1 month
INR	2.04	6.06	0.41	7.59	−0.26	12.76*	−0.56	24.75*	5.6	36.66*	1 year	1 month
APTT (s)	5.56	7.94	4.21	9.6*	4.86	12.13*	9.19	22.64*	18.64*	46.73*	6 months	15 days
TT (s)	0.31	0.62	1.04	1.19	3.11	1.72	2.96	1.41	5.89	8.04	1 year	1 year
Fbg (g/L)	0.79	−0.72	0.8	−0.16	−0.3	0.11	0.72	−0.23	1.11	−0.21	1 year	1 year
FVIII:C (%)	−2.69	−7.9	−7.99	−10.68*	−13.11*	−17.33*	−13.5*	−20.41*	−15.46*	−38.65*	1 month	15 days
FIX:C (%)	−1.71	−3.8	−5.39	−7.92	−12.37*	−18.46*	−31.2*	−34.69*	−34.26*	−37.4*	1 month	1 month

*Changes of >10% in individual samples occurred in >25% of samples.

### Scatter plots

**Figure 1:**
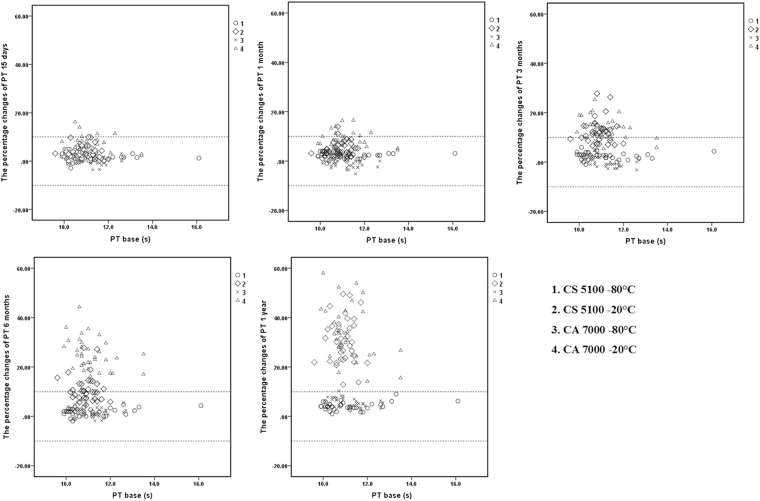
Percent changes of PT in samples stored for 15 days, 1 month, 3 months, 6 months, and 1 year and detected by the CS5100 and CA7000 systems. 1: Samples stored at −80 °C and analysed by the CS5100 system; 2: samples stored at −20 °C and analysed by the CS5100 system; 3: samples stored at −80 °C and analysed by the CA7000 system; 4: samples stored at −20 °C and analysed by the CA7000 system. The two dotted lines in the figures represent plus or minus 10% changes.

**Figure 2:**
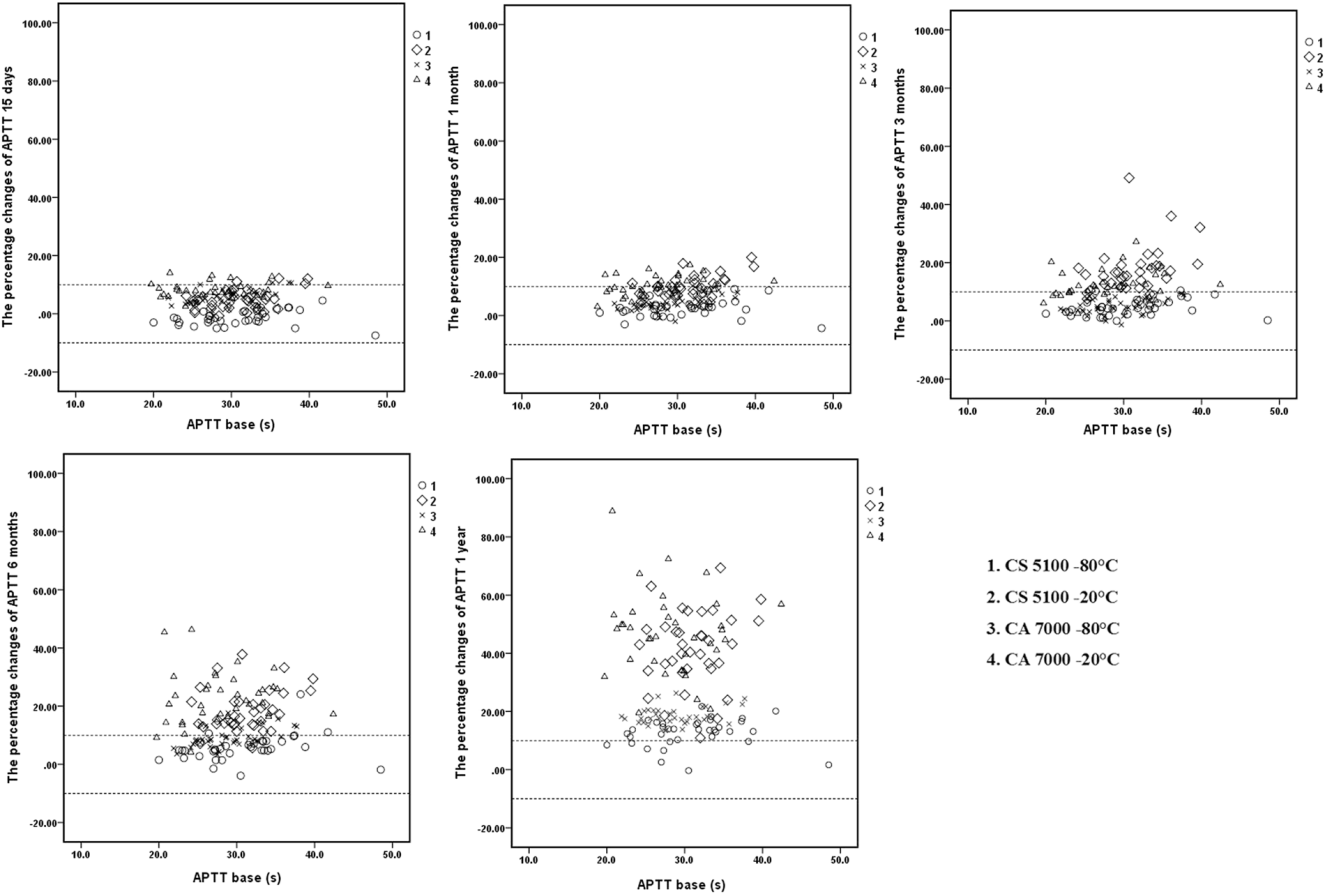
Percent changes of APTT in samples stored for 15 days, 1 month, 3 months, 6 months, and 1 year and detected by the CS5100 and CA7000 systems.1: Samples stored at −80 °C and analysed by the CS5100 system; 2: samples stored at −20 °C and analysed by the CS5100 system; 3: samples stored at −80 °C and analysed by the CA7000 system; 4: samples stored at −20 °C and analysed by the CA7000 system. The two dotted lines in the figures represent plus or minus 10% changes.

**Figure 3:**
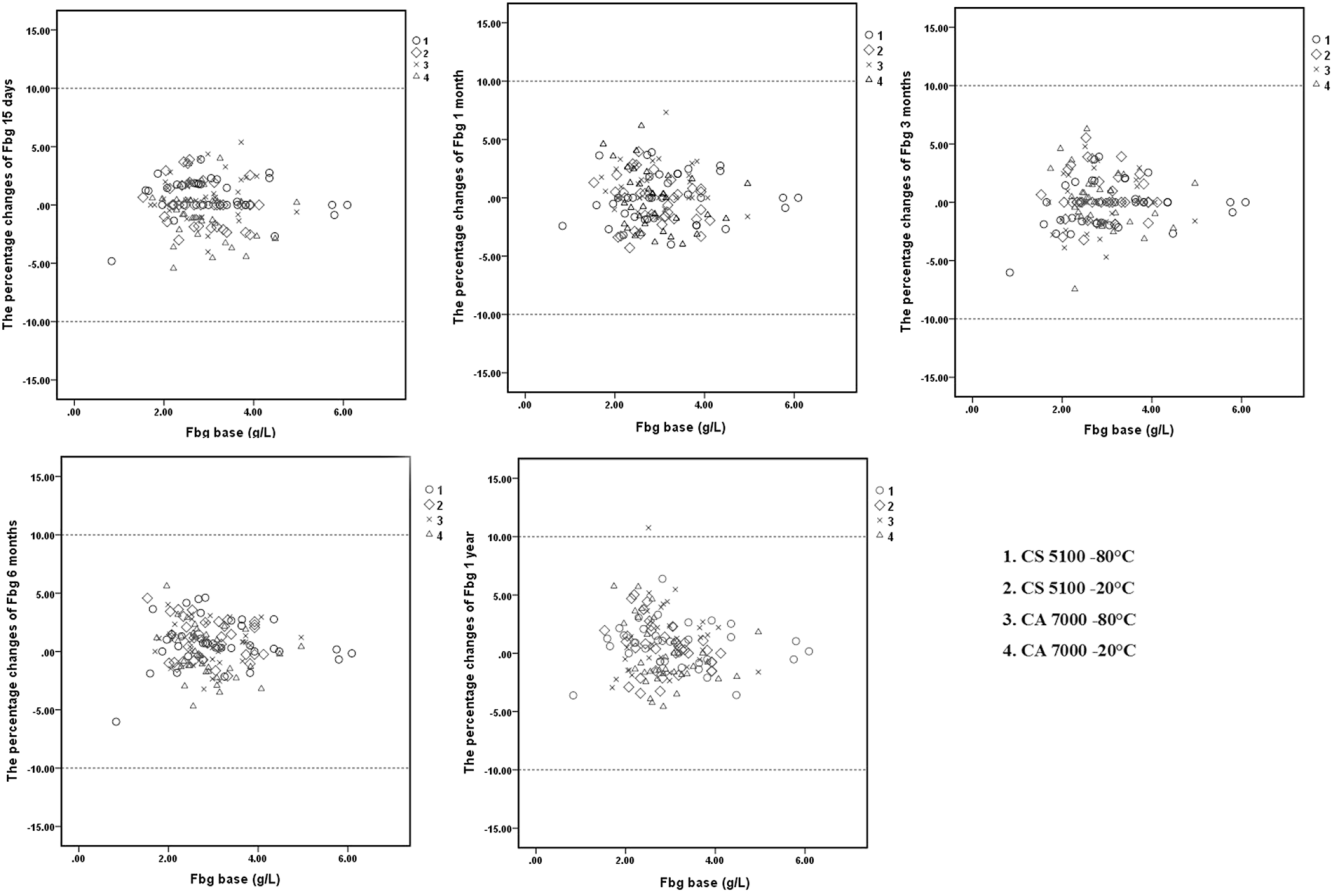
Percent changes of Fbg in samples stored for 15 days, 1 month, 3 months, 6 months, and 1 year and detected by the CS5100 and CA7000 systems. 1: Samples stored at −80 °C and analysed by the CS5100 system; 2: samples stored at −20 °C and analysed by the CS5100 system; 3: samples stored at −80 °C and analysed by the CA7000 system; 4: samples stored at −20 °C and analysed by the CA7000 system. The two dotted lines in the figures represent plus or minus 10% changes.

**Figure 4:**
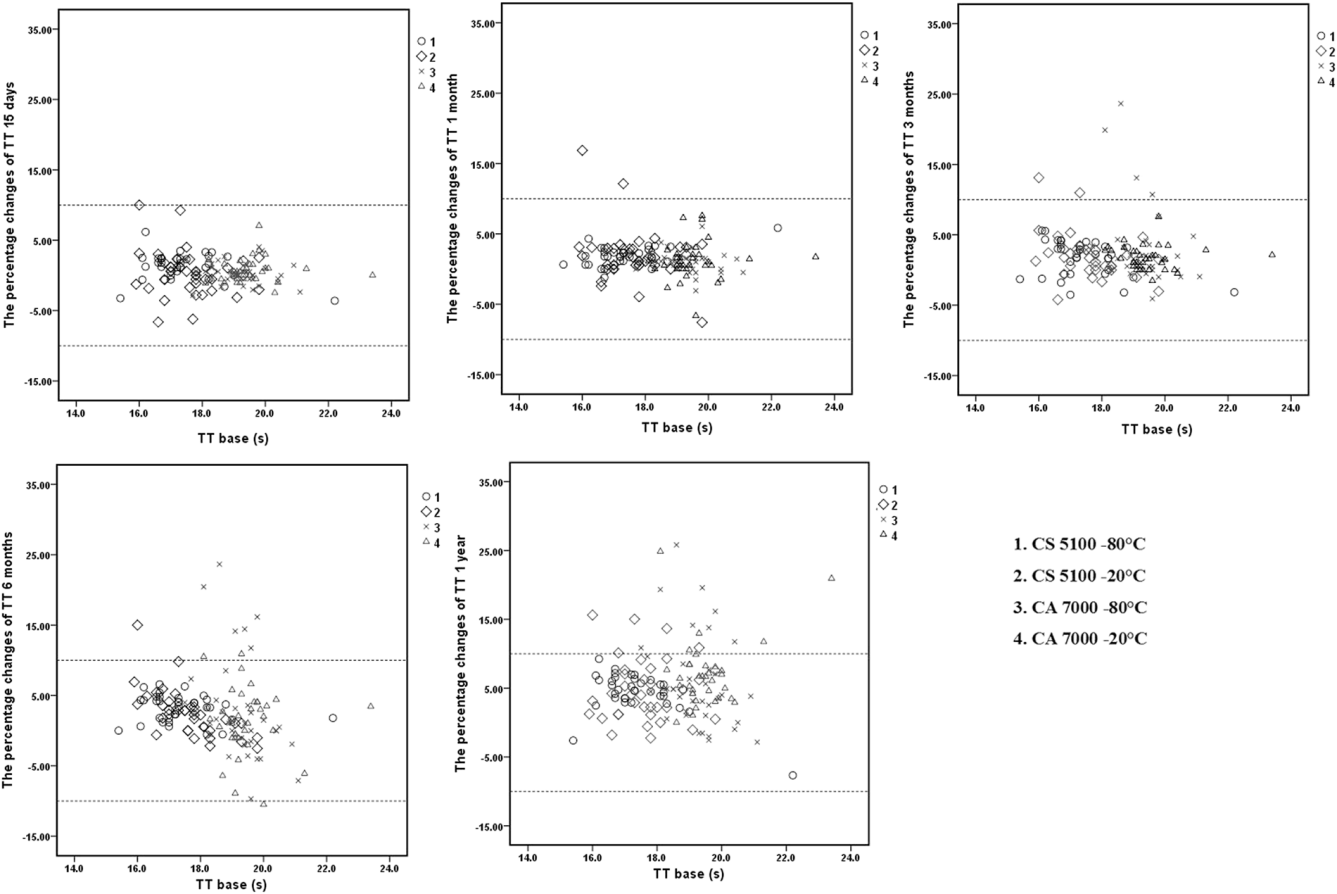
Percent changes of TT in samples stored for 15 days, 1 month, 3 months, 6 months, and 1 year and detected by the CS5100 and CA7000 systems. 1: Samples stored at −80 °C and analysed by the CS5100 system; 2: samples stored at −20 °C and analysed by the CS5100 system; 3: samples stored at −80 °C and analysed by the CA7000 system; 4: samples stored at −20 °C and analysed by the CA7000 system. The two dotted lines in the figures represent plus or minus 10% changes.

**Figure 5:**
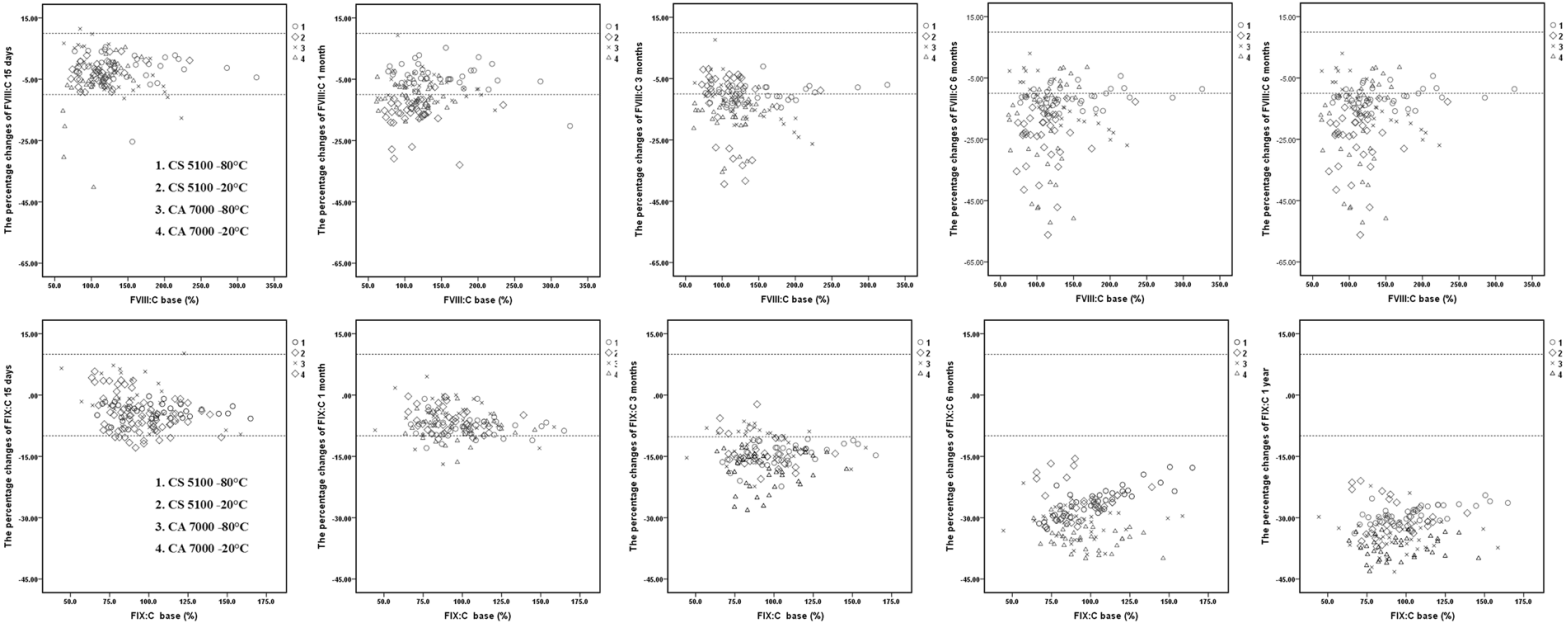
Percent changes of FVIII:C and FIX:C in samples stored for 15 days, 1 month, 3 months, 6 months, and 1 year and detected by the CS5100 and CA7000 systems. 1: Samples stored at −80 °C and analysed by the CS5100 system; 2: samples stored at −20 °C and analysed by the CS5100 system; 3: samples stored at −80 °C and analysed by the CA7000 system; 4: samples stored at −20 °C and analysed by the CA7000 system. The two dotted lines in the figures represent plus or minus 10% changes.

Figures 1, 2, 3, 4 and 5 show the percent changes of PT, APTT, Fbg, TT, FVIII:C, and FIX:C in samples stored for 15 days, 1 month, 3 months, 6 months, and 1 year at −20 °C or −80 °C and measured by the CS5100 or CA7000 systems. The two dotted lines in the figures represent plus or minus 10% changes. Therefore, individual samples with changes of >10% can be visualized in the scatter plots. Furthermore, the numbers of samples with changes of >10% in each group (over nine samples in this study) can be visualized.

## Discussion

Reliable measurements of coagulation function and coagulation factor activity can be affected by sample storage temperature and time from sample collection to testing. We previously found that, along with prolonged time from sample collection to testing, coagulation test times were increased or decreased and coagulation factor activity was significantly reduced at both 4 °C and 25 °C^10^. If samples cannot be analysed in a timely manner according to recommended guidelines, the CLSI suggests that samples should be frozen for later analysis. However, the CLSI does not specify optimal storage times^7^. In this study, we evaluated the effects of freezing time and temperature on screening coagulation tests and coagulation factor activity. According to the mean percent changes and scatter plots, the acceptable stabilities of coagulation tests and factor activities measured by the CS5100 system were consistent with those measured by the CA7000 system at both −80 °C and −20 °C. Therefore, the different coagulation analysers had no significant effect on the quality of the results, although some subtle differences may be observable from the plots. The safe storage times for PT/INR, APTT, and FVIII:C (1 year, 6 months, and 1 month, respectively) at −80 °C were longer than the times (1 month, 15 days, and 15 days, respectively) at −20 °C. The safe storage times for Fbg, TT, and FIX:C (1 year, 1 year, and 1 month, respectively) at −80 °C were the same as those at −20 °C. Although the safe storage times at −80 °C/−20 °C for FVIII:C (1 month/15 days) and FIX:C (1 month/1 month) were longer than the times for FVIII:C (2 h/2 h) and FIX:C (4 h/4 h) at 4 °C/25 °C^10^, their safe storage times were significantly shorter than those for APTT, Fbg, PT/INR, and TT.

Although there are many studies on the effects of freezing storage time and temperature on coagulation function, FVIII:C, and FIX:C, they primarily involved coagulation tests and measurements of coagulation factor activity in FFP after thawing^20,25–28^. FFP is prepared by separating plasma from whole blood within 6 h after donation. The FFP is then frozen and stored at −18 °C or below^27,29^. As early as 1989, Dzik *et al*.^20^ found a greater decline in the factor VIII:C levels in FFP stored for 72 h at −65 °C compared with control plasma immediately after thawing. Philip *et al*.^25^ found that the activity of vitamin K-dependent coagulation factors (FII, FVII, FIX, and FX) and the level of Fbg were within the normal ranges, and adequate for transfusion in twice frozen and thawed FFP stored at −18 °C or below for 1 week. Ben-Tal *et al*.^28^ also found that PT, FVII, FIX, FX, and Fbg were stable and adequate for transfusion in twice frozen and thawed FFP stored at −80 °C for 1 week. Gosselin *et al*.^27^ evaluated the levels of PT, APTT, Fbg, FVIII:C, and FIX:C stored at −70 °C for at least 1 week, and found significant differences in PT, APTT, and FVIII between fresh and frozen plasma. FFP is used to treat bleeding in patients with multiple coagulation factor deficiencies, or for the prevention of surgical bleeding^29,30^. We examined the percent changes of coagulation tests, FVIII:C, and FIX:C, and observed increases in PT and APTT, and significant decreases in FVIII:C and FIX:C. In contrast to our study, the previous researchers primarily aimed to measure the levels of coagulation function and coagulation factor activity. They determined whether coagulation factor activities in FFP were within the normal ranges, and whether FFP could be transfused to avoid wastage. In addition, they did not freeze plasma immediately after blood collection, and the frozen storage time was short.

Other studies have proposed acceptable times for tests of coagulation function and coagulation factor activity in frozen plasma, but not in FFP^26,30^. Woodhams *et al*.^26^ concluded that APTT, TT, PT, Fbg, and coagulation factor activity (allowing for 10% variation) in normal citrate plasma samples were stable for up to 3 months if frozen at −24 °C or below, and stable for at least 18 months if frozen at −74 °C. They found significant prolongation of PT and APTT, reduction of FVIII:C and FIX:C, and no relevant changes of Fbg during storage for up to 24 months. Additionally, they found that the freezing process (freezing at −74 °C and storage at −24 °C vs. freezing at −24 °C and storage at −24 °C) was not responsible for changes in stability. Alesci *et al*.^31^ found that freezing and storage strongly influenced PT and APTT assays and weakly influenced Fbg assays, and showed that the changes in PT, APTT, and Fbg were smaller in samples stored at −70 °C compared with −20 °C after 1, 2, 3, and 4 months of storage. In our study, we aimed to measure coagulation function and coagulation factor activity immediately after blood collection. Furthermore, we aimed to determine suitable storage times and temperatures, and analysed individual differences between samples. Woodhams *et al*.^26^ analysed 600 ml of mixed plasma collected from six healthy volunteers. Furthermore, they did not describe the time period from blood collection to freezing, and did not take individual differences into account. Meanwhile, Alesci *et al*.^31^ did not study changes in FVIII:C and FIX:C. Although the present results were different than those reported by Woodhams *et al*., Alesci *et al*., and our previous study, these studies indicated that the stability of coagulation function and coagulation factor activity in frozen plasma stored at approximately −80 °C was better than that of frozen plasma stored at approximately −20 °C, refrigerated plasma at 4 °C, or fresh plasma at room temperature.

Our study had some limitations. First, only plasma samples obtained from healthy subjects were used. The stability of some coagulation tests and coagulation factor activities may be affected by the clinical states of patients or drugs being taken. In addition, plasma samples with abnormal coagulation function or coagulation factor activity may behave differently. Second, we used only one source of reagents (Siemens) and one type of collection container. Third, our study was a single-centre study. Fourth, we did not study different freezing processes, such as freezing with liquid nitrogen, freezing at −80 °C and storage at −20 °C, or freezing at −20 °C and storage at −80 °C. Our conclusions should be further validated by multicentre studies based on different study populations, reagents, collection containers, and freezing processes.
